# Profitability Optimization of Dairy Farms: The Effect of Pregnancy Rate and Culling Decision

**DOI:** 10.3390/ani14010018

**Published:** 2023-12-20

**Authors:** Violetta Tóth, Emília Heinc, Edit Mikó, Tibor Csendes, Balázs Bánhelyi

**Affiliations:** 1 Faculty of Agriculture, University of Szeged, Andrássy út 15., H-6800 Hódmezővásárhely, Hungary; toth.violetta@szte.hu; 2 Institute of Informatics, University of Szeged, Árpád tér 2., H-6720 Szeged, Hungary; heincze@inf.szte.hu (E.H.); csendes@inf.szte.hu (T.C.); banhelyi@inf.szte.hu (B.B.)

**Keywords:** milk production, culling rate, profitability, microsimulation

## Abstract

**Simple Summary:**

In dairy farms, the culling decision is a complex decision that is influenced by many factors. Economy must also be taken into account when making a decision. The aim of the study was to use a microsimulation to specifically determine the date of culling in addition to the effect of different levels of pregnancy rate on milk production. The results showed that increasing the pregnancy rate successfully reduced the length of the calving interval, but the improved pregnancy rate did not show a significant increase in milk production. Based on this, dairies can more easily determine the time of culling since it has been shown how the milk production of cows can be expected to develop with different pregnancy rates.

**Abstract:**

One of the most important decisions in dairy cattle production today is the correct choice of culling time for cows. In the culling decision process, the farmer has to take into account a number of factors, the complexity of which makes the decision-making task difficult. A crucial factor is the evolution of reproductive indicators. The aim of the research was to develop a microsimulation method that can be used to easily investigate the impact on profitability of increasing pregnancy rates and when the culling decision is made. In the microsimulation, the stock was examined without changing any other conditions. A microsimulation method has been developed to determine with high accuracy the effect of the pregnancy rate and the increase in culling days on the economic indicators of individual dairy farms. By microsimulation, the effect of changing these two parameters on the expected milk production of cows, the most important economic indicator for cattle farms, was investigated. The other parameters of economic importance were simulated using a cattle farm database. The purpose of microsimulation is to assist in producing certain managerial decisions in order to achieve better profitability and economic efficiency. In summary, the results showed that increasing the pregnancy rate can successfully reduce the length of the calving interval, but the improved pregnancy rate did not show a significant increase in milk production. In order to obtain results that can be used by farms, the authors intend to further develop the model in the future, adapting it to farms and taking into account their specificities.

## 1. Introduction

The first calving age and calving interval are important economic indicators for dairy farms [1,2,3]. Several papers have examined the first and second calving intervals, which averaged 412.8 and 599 days, respectively [4,5,6,7]. Toghiani Pozveh [8] and Shadparvar and Ghiasi [9] have shown that the heritability of the calving interval ranges from 0.11 to 0.18. This low heritability value shows that the evolution of the calving interval is strongly influenced by environmental conditions. Thus, improvements in feeding and reproductive management have a greater impact on the calving interval than genetic selection [10]. A key measure of the value of dairy cattle is good reproductive performance, which influences the overall efficiency and profitability of dairy herds. Functional traits, such as fertility, longevity, and health traits, have a significant impact on the efficiency of dairy farming [11].

Several studies have shown that the intensive increase in milk production of dairy cows has a negative impact on health [12] and fertility traits, as there are antagonistic genetic relationships between production and reproductive traits and health status [13,14,15,16]. According to Haile-Mariam et al. [17], if selection is directed to increase milk yield in the first and second lactation, the calving interval will increase. Inadequate reproductive performance of the herd, as reflected, for example, in a longer calving interval, may increase the rate of involuntary culling and result in less milk and fewer calves being produced in a given year [18]. This will reduce the possibility of strategically justified culling, thus increasing the cost of setting up replacements, which will lead to lower net returns [19]. Melendez and Pinedo [16] evaluated 150,457 lactation data from 1990 to 2003, and they found that, for every 100 kg milk yield standardised to 305 days, the calving to conception interval increased by 0.6 days and the first insemination success decreased by 0.9%. Reproductive biology disorders cause economic losses in two ways. The first is the loss of milk production due to a prolonged calving interval, and the second is the increased cost of replacements due to fewer calves per cow [20,21,22,23]. However, some studies suggest that a longer calving interval is more beneficial. According to Niozas et al. [24] fertility outcomes can be improved and fewer inseminations are needed if the first insemination is postponed from 40 to 120 days after calving because the metabolic status of cows is better at this time. In addition, extending the calving interval can reduce the number of cows with high milk yields prior to dry-off (i.e., >18 kg/day), which can improve udder health [25,26] and cow welfare [27], as milk yields of high-yielding cows at drying off often exceed 18 kg at the 1-year calving interval, which increases the risk of udder infection during drying off and post-calving [26].

According to Burgers et al. [28], extending the voluntary waiting period of first insemination after calving can elongate the calving interval, which can have a positive impact on fertility and health, as the dry period, calving, and the onset of new lactation are critical transition events in the life of dairy cows. These changes increase the risk of disease and culling [29,30,31]. A 1-year calving interval is associated with a high average 305-day milk yield and better economic outcomes compared to a longer calving interval, as most studies that retrospectively analysed data from cowsheds found that cows with a longer calving interval had lower annual milk yields compared to cows with a shorter calving interval, but this milk loss was not significant [32,33,34,35,36].

The negative effects of pregnancy on milk yield can be delayed if the calving interval is extended, increasing the persistence of lactation [37,38]. At longer calving intervals, heifers showed no or a lower milk yield loss than cows that calved more than once [39,40,41]. This was also found in studies by Burgers et al. [42] in heifers, with no effect on daily milk yield when the voluntary waiting period was extended to 200 days. However, for multiparous cows, a voluntary waiting period of 200 days resulted in reduced daily milk yield and increased body condition scores at the end of lactation and in the following lactation, compared to multiparous cows with a voluntary waiting period of 50 days. Some studies reported lower services per conception [24,43] and better pregnancy rates [24,34,43] when the voluntary waiting time after calving was increased. In studies by Burgers et al., the persistence of lactation increased in most cases with longer calving intervals [28]. Heifers showed the highest effective lactation yield when the number of days from calving to the first insemination was 196 or more. And for multiparous cows, the highest actual milk yield was obtained when the number of days from calving to first insemination was more than 140. In a study by Burgers et al., in the last 6 weeks before calving, cows that were first inseminated at 125 days after calving had lower milk yields than cows that were inseminated at day 50 after calving [42].

Österman and Bertilsson [40] found that lactation was 20.4 weeks longer in the 18-month calving interval than the 12-month calving interval. At the 12-month calving interval, milk production was 6.2 kg at the beginning of the dry period and 0.7 kg at the 18-month calving interval. The 18-month calving interval resulted in an increase of 1.3 energy-corrected milk, hereafter ECM, kg/day for primiparous cows and a decrease of 1.0 ECM kg/day for multiparous cows compared to the 12-month calving interval. This experiment shows that an 18-month calving interval combined with three milkings per day results in the highest ECM/day. For the 12-month calving interval, cows were first inseminated after 50 days, and the milk lactation curve began to decline steeply around week 30. For the 18 month calving interval, cows were first inseminated after 230 days, so milk production declined around week 55. Ratnayake et al. [44] reported that second-calving cows required less artificial insemination at 15- and 18-month calving intervals than at a 12-month calving interval. It has also been shown that increasing the calving interval to 18 months can have a positive effect on reproductive indicators by reducing the need to treat ovarian abnormalities and increasing conception rates. The results of Arbel et al. [45] suggest that a longer calving interval is preferable for primiparous cows than for multiparous cows. According to García et al. [46], infertile insemination was 3.9 times higher in cows inseminated in the warmer months (May to September) than in those inseminated in the cooler months (October to April). De Rensis and Scaramuzzi [47] have shown that fertility in a dairy herd can be up to 20–30% worse in the summer than in the winter months. This is confirmed by a study by Nardone et al. [48], who described a 20–27% reduction in conception rates during the summer. The aim of the research was to accurately determine the culling date from an economic point of view by developing a microsimulation model based on pregnancy rate and milk production parameters.

## 2. Materials and Methods

### 2.1. The Investigated Dairy Farms and the Method of Data Collection

Data from six dairy farms in southern Hungary were used to build the simulation. The parameters analysed were collected from the herd management system (RISKA). All dairy farms had Holstein Friesian cows; herd sizes varied between 400 and 1400. The average daily milk production was between 34 and 39 kg. The average calving interval ranged from 411 to 462 days on the surveyed dairy farms. The average number of lactations was 1.9–2.2. Cows were milked three times a day on each farm. The husbandry of productive cows was different. There were farms where straw was used in the barn, and there were farms where the cows were housed in laying boxes with rubber mattresses. Each farm used a neck- or leg-mounted transponder to collect reproductive biology data, such as the time of oestrus and insemination. For accurate analysis, farms where no synchronisation of oestrus was performed were included in the experiment. Artificial insemination was used on all farms. The following data were available: the time of first insemination after calving, the time between two unsuccessful inseminations, the length of gestation, calving interval (in days), and the success of inseminations. The period between 1980 and 2020 is included in the study. It was only after processing and analysing these data that the right simulation could be created. Custom-made Python codes were applied for the statistical data processing and distribution fitting. This was necessary because, by providing the microsimulation with a configuration file containing the continuous distributions fitted to the real data, the simulation can stochastically generate the simulated cow data according to the continuous distribution.

### 2.2. Choosing a Statistical Model

For cases not found in the literature, we needed to select from the fitted distributions the distribution that best fit the extracted sample using the maximum likelihood method [49]. To achieve this, we needed a metric that gives the “goodness” of each distribution. Based on these values, we could develop a ranking of which distribution best fit our data. We examined which of the given distributions best fit the data by several metrics.

The first such metric we used was the Akaike information criterion (AIC) [50], which, using the loglikelihood value, favours simpler distributions with fewer parameters according to Occam’s razor, i.e., it uses a penalty function equal to the number of parameters.

This metric penalises overfitting, since continuous distributions with a smaller number of parameters are usually not overfitted.

To achieve this, we fitted all continuous distributions to the resulting sample and then ranked the distributions in ascending order by AIC value.

Among the other metrics we examined is the Bayesian information criterion (BIC) metric [51], which is very similar to the AIC metric. This metric also penalises distributions with a large number of parameters. The difference is in the weighting, which also depends on the dataset.

The third metric examined was the sum of squared differences between the density function of the fitted distribution and the probability density of the real data for each data point.

These mods were used to find the best distribution function for the random events that were used in the modeling. These random events are shown in the following chapters.

### 2.3. The Distribution of the Calving to First Service Interval (CFSI)

One of the most important variables influencing the length of lactation in cows, the calving to first service interval (CFSI), was investigated. The following characteristics had to be taken into account when processing the data on the dates of the first heating after calving: we had a biased distribution since artificial insemination was applied; the distribution is also distorted by the fact that the cow’s oestrus is sometimes detected late, thus delaying the CFSI; smaller data values in the database were erroneously recorded as the first oestrus dates, which also had to be dealt with. Accordingly, we transformed the data by truncating the smallest values by 1% and then fitted the distributions to them:

- Johnson su: $f\left(x\right)=\frac{b}{\sqrt{x^{2}+1}}\phi \left(a+b\log\left(x+\sqrt{x^{2}+1}\right)\right)$
- genextreme: $f\left(x\right)=\exp\left(-{\left(1-cx\right)}^{1/c}\right){\left(1-cx\right)}^{1/c-1})$
- mielke: $f\left(x\right)=\frac{kx^{k-1}}{{\left(1+x^{s}\right)}^{1+k/s}}$
- burr: $f\left(x\right)=cd\frac{x^{-c-1}}{{\left(1+x^{-c}\right)}^{d+1}}$
- invgamma: $f\left(x\right)=\frac{x^{-a-1}}{\Gamma (a)}\exp\left(-\frac{1}{x}\right).$

**Figure 1:**
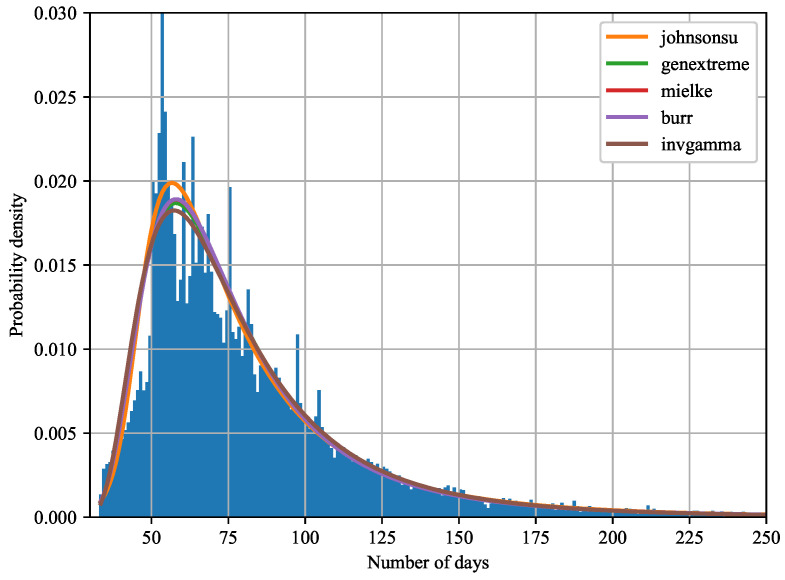
Plot of the evolution of the CFSI as a function of density and the first five distributions fitted by the AIC value.

The resulting top five distributions by AIC value are given in Figure 1. They are plotted against the real data. The other distributions are compared in the following table based on the three metrics listed. The top five distributions are shown in Table 1.

The best distribution based on all three metrics is the Johnson $S_{U}$ distribution. The distribution is the inverse hyperbolic transform of the normal distribution [52]. In reality, all natural events usually follow a normal distribution. In this case, this distribution has been distorted because the artificial interventions have made the distribution of the real data asymmetric. The other factor that distorted our distribution was that there were silent heating cows, so in their case, the date of first insemination was delayed.

The Johnson distribution has also become the best fit for our data because it is a transform of the normal distribution and it can fit asymmetric, fat-tailed distributions, such as the real distribution, flexibly [53].

### 2.4. Length of Gestation

The length of gestation follows a normal distribution in reality. According to the literature, the gestation period of a cow is between 260 and 300 days. We left the real data between these two values and fitted the distributions to them.

**Figure 2:**
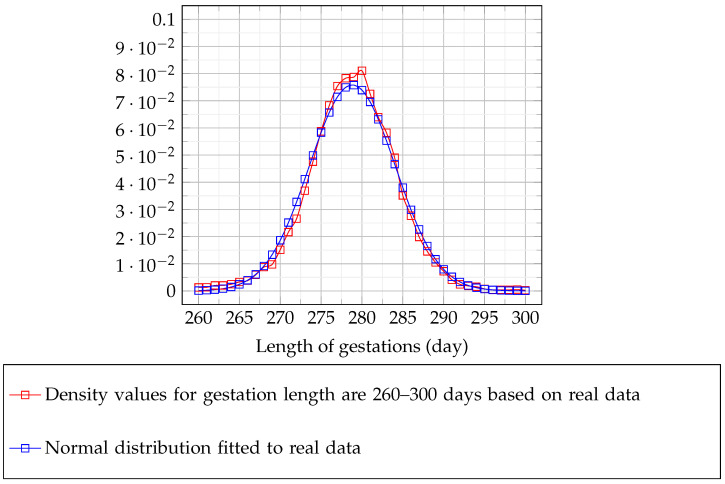
Comparison of exact expected values and approximate simulation results.

**Figure 3:**
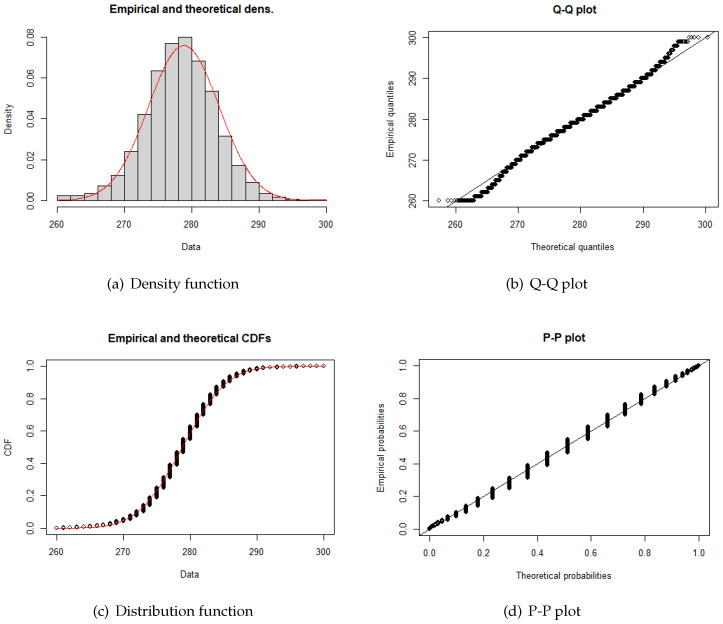
Normal distribution fitted to real data on gestation length (the markers represent the real data, while the solid lines represent the fitted data series).

Since our data volume is greater than 5000, it is not recommended to perform a statistical test on the hypothesis that our distribution follows a normal distribution [54]. The larger the amount of data, the more sensitive the statistical tests will be to the noise and discrepancies in the data. Because the length of gestation of the cow follows a normal distribution [55], a bell curve was fitted to the data (see Figure 2 and Figure 3).

This led us to examine the Q-Q and P-P plots of the fitted curve. Of these, an examination of the Q-Q diagram answered the question. Both P-P and Q-Q plots compare a set of data with a theoretical distribution function. In the case of P-P, it compares the cumulative distribution functions, while in the case of the Q-Q diagram, it compares its quantiles. Based on the Q-Q diagram, there is a difference between the expected and the theoretical values at the extreme values of the cut-off distribution. This is due to the not-entirely accurate truncation of the interval to the length of gestation.

### 2.5. Time between Successive Inseminations

The basic oestrus cycle of the cow averages 21 days in duration. As with the first insemination, in the case of late-onset oestrus, we try insemination again after the next 21 days. Late detection of oestrus may be repeated several times. This makes the distribution of the real data a distribution with several local maxima. When fitting the curve, it is not suitable to fit a known continuous distribution curve, but it is necessary to use some kind of mixed model to cover the important local maxima.

**Figure 4:**
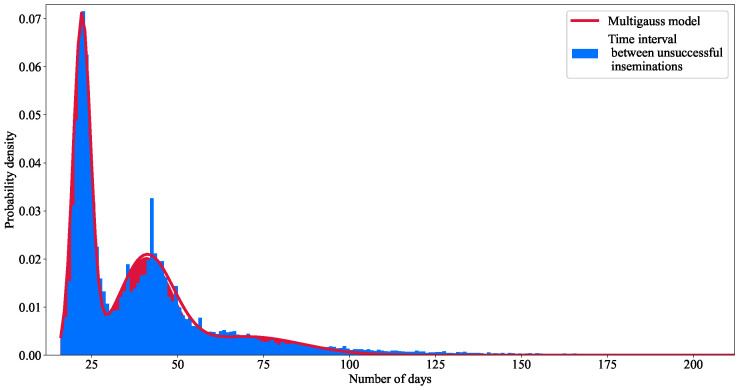
Real and multi-Gaussian model fitting of time intervals between unsuccessful inseminations.

A mixed Gaussian model was therefore fitted to the data using an expectation–maximisation (EM) algorithm [56]. An EM algorithm is an iterative method to find maximum likelihood estimates of parameters in statistical models. We first fit five normal distributions to the curve. The results are summarised in Table 2. The result of the fitting is shown in Figure 4.

To prevent overfitting, as large values in the database may indicate erroneous data and the weights of the last two distributions with the largest mean values are negligibly small (less than 0.05), the last two distributions can be dropped from the model. In what follows, we kept only the first three of the five fitted distributions.

### 2.6. Effective Fertility

**Figure 5:**
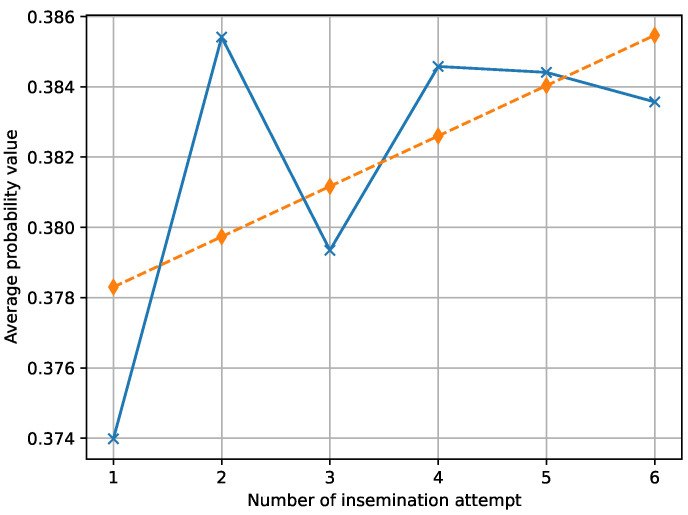
Probability density values calculated for the pregnant herd only and for the whole herd as a function of the number of inseminations (the solid line is the real data, the dashed line is the linear fitted data).

For the largest herd, we tested whether the pregnancy percentage was independent of the number of inseminations. When looking at cows that were known to have been pregnant (i.e., not culled without insemination), the average pregnancy percentage was 38.1%, regardless of the number of inseminations. The dependency/independency of the line number is shown in the first part of Figure 5.

As can be seen, the linear correlation R is small ($R^{2}=0.36$), as is the correlation coefficient. That is, in our simulation, we consider the conception rate (CR) to be independent of this sequence number.

In fact, for all cows, the pregnancy rate in our herd is much lower because there are cows that will never become pregnant. For example, they are culled or unable to conceive for biological reasons that are not visible to the keepers. Since their culling is not perfect, the percentage of pregnancies will show a small decrease due to these cows. See the second part of Figure 5. We did not deal with culling and its accuracy in the simulation, so we compared everything to the previous data. Similar independence was observed in the other five dairy farms.

The average success rate was calculated from data from the largest site and had a constant value of 0.28, which was obtained by taking all the insemination attempts that occurred on the dairy farm (17 insemination attempts were the most) and calculating the percentage of successful inseminations from the total attempts. This value is a good approximation of the expected value of pregnancy success. The simulation simply generates a pseudorandom number between 0 and 1, and if it generates a value greater than a given constant for a given insemination attempt event, then we say that the insemination has failed, otherwise it has succeeded. A careful application of a microsimulation enabled us to investigate complex models when the necessary data are available in the form of empirical distributions or at least histograms, and we wanted to understand the consequences of changes in decision protocols applied to the underlying statistical model.

### 2.7. Model Verification

Along with the distributions given in this chapter, a cow’s lifespan could be generated using a microsimulation method. In the simulation configuration, you can specify not only constant values, but also the specific data and the types of the selected fitted distributions. Based on the input configuration, the simulation builds a stochastic model consisting of a chain of specified events. Events are now not given constant values as time intervals to occur interdependently but are pseudorandom numbers generated by a given distribution.

In order to use the method to determine more correlations about the cow’s life cycle, we had to see if the simulation worked correctly.

The configuration of the simulation was modified to change the cessation of milk production from 200 days to 300 days. The simulation was run on a total of 1000 cows, not all of which were successfully inseminated. The simulation was configured so that the cull condition, when the simulation is stopped, is the day the cow stops producing milk or when the next lactation of the cow begins, i.e., the first day after calving. We calculated the time interval for each cow from when we started the simulation until calving occurred. If this event did not occur, it was not included in the statistics. That is, the time period was tied to the occurrence of an event. In total, there were 880 successful inseminations. From the real data, time intervals longer than 600 days were irrelevant and were cut from the data.

**Figure 6:**
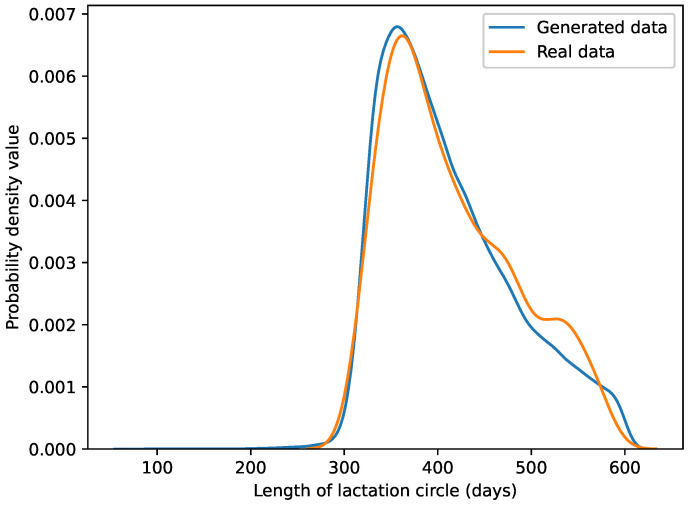
Comparing the probability densities of real and generated data.

The resulting two datasets were compared and are shown in Figure 6. The comparison was performed with different statistical tests at a 0.05 significance level.

The first statistical test we used was the two-sample Kolmogorov–Smirnov test, the null hypothesis of which was that the two samples, the generated and real data, were derived from a theoretical distribution [57]. The test takes as the distribution function of the two samples to be compared, at each discrete point, the absolute value of the difference between the two samples. If this value is greater than a given significance level and sample size, the hypothesis is rejected. At a significance level of 5%, the *p* value of the test is 0.123, which means that the hypothesis is not rejected.

We also tested the hypothesis that the means of the two samples are the same using a two-sample *t*-test at a significance level of 5%. With a *p* value of 0.055, i.e., p>0.05, the null hypothesis is not rejected, which means the two distributions are equal [58].

As the culling decision can be both compulsory and voluntary, in practice, in many cases, the protocol of the farms for culling cows on a given day is not always consistent. In practice, the timing of the culling decision is mostly determined by the cow’s milk production, reproductive biology, and health status. These factors can override a given site’s culling protocol, causing a minimal difference between reality and the simulated data.

In the following, based on the correctness of the distribution, additional data can be derived by changing the parameters. We can look at the impact of different values on the cow’s life cycle [59,60].

The length of lactation greatly affects the value of the final profit, as there is a dry period in the last 60 days of pregnancy and this means a loss of income. In reality, it is more optimal for dairy farmers to make the cow pregnant as early as possible, thus shortening the length of lactation, as the cow’s milk yield varies throughout her life. Immediately after calving, the cow produces the most milk and gives the highest profit value [61].

## 3. Results and Discussion

### 3.1. Model Parameters

The primary investigated parameter is the fertility success Rate. So far in the cow’s life cycle, the average value has been taken as the fertility success rate. In reality, this value changes every month. It can be observed that the proportion of cows that become pregnant in the summer is much lower than at other times of the year. The simulation has therefore been extended to take into account the month in which insemination occurs. In total, data were taken from six different dairy farms, and so far, only one farm has been examined. The simulation was further configured so that the success of conception depends on the month in which it occurs.

Monthly fertility success rates were calculated from averages. The average success rates are shown in Table 3, broken down by month. The table shows the average conception rate per month, calculated on the basis of data from six dairy farms. These values were taken as an approximation of the expected success rate and used to calculate the fertility success rate. According to this table, the calving intervals were generated for each dairy farm, taking months into account.

Table 3 shows the average monthly pregnancy results of the six examined large-scale dairy farms in Hungary. In all cases, the months indicate the month of artificial insemination. Significant differences can be observed between the farms’ annual pregnancy results; the average annual pregnancy rate varied between 26.5 and 42.5%. Of the six investigated farms, the pregnancy indicators of the first farm proved to be the weakest. The highest pregnancy rate on this farm was around 32%, which occurred in the month of November. On this farm, the minimum pregnancy rate was recorded in July, reaching only 17%. In terms of pregnancy rate, the two most successful farms were farms 2 and 5. In the case of these farms, the maximum pregnancy rate reached up to 46%. Overall, there was very little variation in average monthly pregnancy results between these two farms, indicating a high-quality and tightly managed breeding programme. From the data in the table, it can be seen that the pregnancy rate was the lowest for all farms in the period from June to September. Rensis et al. [62] observed similarly reduced fertility in the Northern Hemisphere during the same period. In terms of the pregnancy rate, the period from October to May proved to be the most favourable. Of course, it must be kept in mind that the reproduction indicators of a large-scale dairy farm are influenced by many factors, such as management, the level of milk production, husbandry technology, feeding, animal welfare, and the animal health status of the farm, and a dairy farm must be judged in this light. By determining the expected pregnancy rate, the microsimulation model developed during the research helps to more easily determine the selection of the culling date, since the two most important factors in making a voluntary culling decision are usually the quality of the cow’s milk production and her reproductive biology status. Pinedo et al. [63] highlighted in their study that successful reproduction and high milk yield are associated with a lower culling risk. By taking these factors into account, the simulation model, as a decision support system, shows us the optimal time for culling. In all cases, it is important to emphasise that farm management can change and influence these parameters and, thus, decisions. One of the parameters used in the study is the pregnancy rate, which is, of course, not a direct decision but can be influenced by farm management.

The other decision in the investigation is the farm culling decision. This was set for the day after the calving. Cows were culled if they had not calved by that date. It is a simple farm decision. Based on this decision, cows with poor milk production and non-pregnant cows are culled.

### 3.2. Profitability

**Figure 7:**
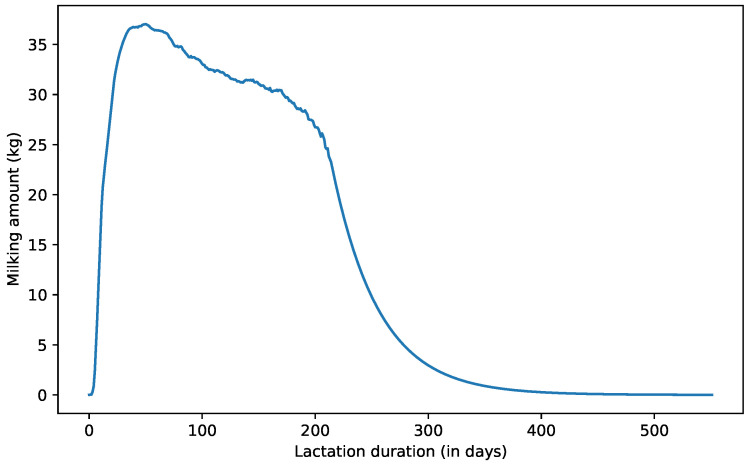
Estimated lactation curve simulated for the six farms.

From the point of view of the farm’s profitability, the most important key issue is the level of milk production of cows. The amount of milk given by a cow depends, to a large extent, on the keeping habits of the dairy farm and especially on the feeding habits. During the study, the average milk production of the six dairy farms was calculated as a function of the days since calving. This is shown in Figure 7. It is clear that the reduction in milk volume is expected to occur on day 217 of lactation. Based on the lactation curve, it can be said that after the 249th day, the amount of milk produced does not even reach 10 kg.

### 3.3. Milk Yield Trends

Running the simulation on 1000 cows, we examined the significant data of several different cows from different farms, depending on the conception rates and the decrease in milk production. After running the simulation under different configurations, the data are shown in Table 4. The simulation is configured so that culling of the cow occurs when she is not pregnant or when her milk production is reduced. Since the length of lactation is the main determinant of milk yield, the simulation is only run until the first calving. This makes the simulation data identical to the lactation cycle data. The typical data for lactation cycles of pregnant cows are shown in Table 5. In the configuration of the simulation, real data from the site were entered, and the previously selected continuous distribution curves were fitted to the real data. The only change to the previous configuration is that there is no dry period 60–70 days before calving but on day 60 of the 280-day average gestation length, i.e., day 215 after conception. When performing the statistical calculations, for the sake of simplicity, a gestation period of 275 days and a dry period of 60 days were calculated. Of course, this can be 285 days in reality, and the dry period can also be extended due to individual effects. In the simulation, the date of the culling decision and the pregnancy rate were investigated for the whole herd (1000 cows) and specifically for cows that had successfully conceived.

### 3.4. Considering All Simulated Individuals

The simulation model developed is able to predict how milk production results will be at different pregnancy rates and culling days based on data from a given dairy farm. The model helps to improve the objectivity of the decision-making process, as the culling decision is usually made subjectively.

In calculating the milk yield for a full herd, the whole herd included in the simulation is taken into account: 1000 cows in total. The studied 1000 cows were simulated according to good practice statistical principles; they were not selected from real cows. This includes animals that have not conceived and have reduced milk production after a certain day. We now consider two unknown parameters: the conception rate and the date on which milk production ceases from the last calving. The data are summarised in Table 4.

The total amount of milk is the value of the total milk yield calculated on the given farm. The milk yields of the dairy farm within the lactation cycle were available. It can be built into the simulation to generate exactly how many cows give milk each day during the lactation cycle. Multiplying this by the actual milk yield over the cycle, we obtain the milk yield we had on the farm. Since the length of lactation cycles decreases in direct proportion to the increase in the success rate, there will be fewer days of milk per cow. This results in both the total milk yield and the total number of days of milk decreasing as the success rate increases. By increasing the time interval before the cessation of milk production, non-pregnant animals also give milk for more time during the given days. The effect of this is that the total amount of milk increases as the day of decline in milk production is extended.

The average length of lactation is the number of days when the milking event occurs for the whole herd. By increasing the success rate, the length of lactation cycles is shortened, so the milking event occurs in fewer days, and by increasing the time at which milk production ceases, milk production in animals that have failed fertilisation attempts lasts longer. The timing of the decline in milk production also affects the number of insemination attempts, as in this case we try to inseminate until the cow’s milk is reduced. In several experiments, more cows will become pregnant.

The average daily milk yield is the ratio of the total amount of milk and the number of days divided by milk yield values. This value is the average milk yield per day calculated when produced by the cow on that day. Figure 7 shows the average milk yields on each day within a lactation cycle. The cows produce most milk in the first days of the lactation cycle. As the days go by, this amount of milk gradually decreases. The figure shows an increase in milk yield at about day 600 because the farm begins culling less profitable animals. As the number of days of reduced milk production increases, the average daily milk yield decreases, because in the case of longer milk production cycles, milk yield decreases as the number of days increases and the average daily milk yield is lower. As the conception rate increases, the average daily milk yield increases as the average length of lactation cycles decreases, and the average milk yield calculated for each day increases due to the daily development of milk yields.

As shown in Table 4, using the model, assume that only 20, 30, 40, or 50% of 1000 cows at each insemination become pregnant. Insemination is attempted for 300, 350, or 400 days, if the cow does not become pregnant by this time, they do not attempt further insemination but cull the individual. Using the model, it can be calculated that, at a pregnancy rate of 20%, more milk is expected overall, as the cow will produce milk for more days due to the later pregnancy. As a result, the length of lactation is extended more than with a 50% pregnancy rate, due to the fact that the pregnancy occurs earlier in these cows. Thus, these cows produce milk for fewer days on average (e.g., about 324 days).

Two of the most important factors determining the economics of cattle production are the achievement of good on-farm reproductive biology and the calving interval. Shorter calving intervals can result in more calves born each year and higher milk production without increasing fixed costs [64]. During the study, by increasing the pregnancy rate, the calving interval was successfully reduced. With a 50% pregnancy rate, this period can be reduced to an average of 389 to 395 days. In Hungary, the average calving interval was 423 days, and the average number of milking days was 298 days in 2020 (National Association Of Hungarian Holstein Friesian Breeders, 2020) [65]. In Vargas et al. [66], the average length of lactation was 328 days. López-Gatius [67] found in his study that the pregnancy rate at first insemination varied between 27 and 44%. According to Borbély et al. [68], the profitability of milk production is crucially influenced by the length of lactation. The length of lactation is basically determined by successful insemination. Borbély et al. [68] draw attention to the fact that it is an economically incorrect decision to narrow down one production cycle to lactation, since the production cycle forms a unit with the dry period. During the dry period, the cow does not produce a real saleable yield, so the costs of keeping the cow must be met from production during lactation. Based on these, a production cycle of cows was interpreted. Table 5 shows how the amount of milk between two calvings changes daily with different levels of pregnancy rates. As can be seen in the table, if, for example, the cow is culled on the 400th day and the pregnancy rate is 20%, then this amount of milk is 16.5 kg, and if the pregnancy rate is 50%, then it is 18.7 kg. In our case, increasing the pregnancy rate did not result in an economically significant increase in milk yield. In the study by Von Krueger and Heuwieser [69], the pregnancy rate of the experimental group was 54.8%, compared to 58.2% in the control group. Fodor et al. [70] examined the reproductive biological indicators of 34 large-scale dairy farms, and found that 26.52% of the cows became pregnant at the first insemination after calving.

### 3.5. Considering Only Pregnant Individuals

Only the development of milk yield during lactation cycles was examined separately. From the results presented earlier, we subtracted the values of the individuals that did not become pregnant. So, here we look at the data of the herd that went through the calving event and ran the simulation from calving to calving. Of the parameters examined in Table 5, the explanations for total amount of milk, average length of lactation, and average daily milk yield are the same as those examined in Table 4 and are described above.

The number of cows in lactation indicates how many of all the cows in the simulation run of 1000 cows were successfully inseminated. This value increases with both the success rate and the time at which the milk yield is reduced. In the first case, this is because, on average, the same number of maximum insemination attempts is more likely to result in one successful attempt. And in the second case, this is because we will have more insemination attempts while the pregnancy rate remains the same.

The average calving interval (days) is the number of days from one calving to the next. It decreases in direct proportion to the increase in the pregnancy rate. If we increase the number of days when the cow’s milk is reduced, then more insemination attempts are possible if insemination has failed. This increases the number of days between calvings.

The milk yield calculated for the days between two calvings is calculated by the average daily milk yield for the days of the calving interval.

Table 5 illustrates the expected milk production results of the actual pregnant cows as a function of the culling day. It can be seen that, with a given pregnancy rate, the decision to cull the cow on the 300th or 400th day is not really important from the point of view of milk production, since there is no significant difference in production if the cow is culled after 300 or 400 days. The increase shown in the table is due to an increase in the number of cows, as cows continue to become pregnant. Since the milk production of the cows is then not so high, it is economically worth considering whether the income from the milk produced will cover, for example, the cost of keeping and feeding a cow that is milked for 400 days. When comparing the pregnancy rates with the results of cows culled on the same day, it is also found that there is no significant difference in milk production between the 20% and 50% pregnancy rates. It can be seen that the length of lactation, i.e., the number of milk-producing days, can be extended by the decision to cull later, but that there is a steady decline in daily milk yield at both 20% and 50% gestation rates. Increasing the pregnancy rate had a positive effect only on the calving interval among the parameters studied. At a 50% pregnancy rate and culling at day 300, the calving interval can be reduced to less than 400 days. As we have already discussed, the decision to limit a cycle to only those days when the cow actually produces milk is economically incorrect. The cow makes no real profit during the dry period but is an expense to the farmer, so the cost of the dry period must be earned during her lactation. Based on this, we interpreted one cycle of the cow. It is clear from the table that, if we calculate the daily milk yield per day in the calving interval, there is no significant increase in milk yield by increasing the pregnancy rate or by postponing the day of culling.

## 4. Conclusions

Based on our results, we conclude that it is not milk yield that is primarily affected by increasing the pregnancy rate (the results show that there is little difference in milk yield), but that it provides a greater opportunity for selection. Our results show that increasing the pregnancy rate can successfully reduce the length of the calving interval, but the improved pregnancy rate does not show a significant increase in milk production. Practical experience shows that most voluntary culling decisions are made because of insufficient milk production. One methodological improvement was made during the research. A proprietary model has been developed that works with microsimulation and is able to take into account the context of cow herds. This simulation was used to investigate two parameters: the pregnancy rate and the day of lactation when the culling decision should be made. We also observed the effect of changes in these two parameters on the number of herds. Of course, this simulation must always be adapted to the specific characteristics and circumstances of dairy farms. 

## Figures and Tables

**Table 1:** Comparison of the first five distributions fitted to the first insemination data.

	Distribution	AIC Value	Distribution	BIC Value	Distribution	Sum of Squared Differences
1	Johnson su	301,879.4457	Johnson su	301,912.905	Johnson su	0.002377
2	genextreme	302,003.1927	genextreme	302,028.2872	exponnorm	0.002388
3	mielke	302,009.5675	mielke	302,043.0269	burr12	0.002433
4	burr	302,010.1332	burr	302,043.5925	invweibull	0.002488
5	invgamma	302,040.148	invgamma	302,065.2425	burr	0.002495

**Table 2:** Mixed Gaussian model with K = 5 parameters of the fitted curves obtained for each case.

Distribution	Weights	Averages	Standard Deviations
1.	0.50	22.32	2.62
2.	0.32	41.68	9.03
3.	0.14	72.75	16.07
4.	0.03	126.02	30.68
5.	0.01	208.51	94.69

**Table 3:** Monthly average success rate in the 6 dairy farms.

	1. Farm	2. Farm	3. Farm	4. Farm	5. Farm	6. Farm
January	0.290288	0.427578	0.362306	0.320567	0.460518	0.320567
February	0.297108	0.40757	0.363591	0.326293	0.445264	0.326293
March	0.271527	0.416369	0.372992	0.315096	0.443843	0.315096
April	0.309963	0.390013	0.363683	0.308043	0.446775	0.308043
May	0.272847	0.346596	0.334951	0.293084	0.439539	0.293084
June	0.23063	0.340537	0.310934	0.253014	0.391304	0.253014
July	0.173338	0.336765	0.246849	0.224691	0.360628	0.224691
August	0.187857	0.32552	0.259133	0.225623	0.392649	0.225623
September	0.22706	0.36502	0.31304	0.248262	0.409018	0.248262
October	0.299523	0.383095	0.381153	0.28819	0.444978	0.288103
November	0.320073	0.405457	0.369177	0.299295	0.432553	0.299295
December	0.302183	0.395085	0.369365	0.294806	0.440601	0.294806

**Table 4:** Milk yield data for all herds as a function of success rate (20–50%) and milk production decline (300 days–400 days).

	Day	Successful Insemination Experiment			
		20%	30%	40%	50%
Total amount of milk (kg)	300	7,403,579	7,396,329	7,383,544	7,370,866
	350	7,427,995	7,411,954	7,393,707	7,378,133
	400	7,432,324	7,410,541	7,389,948	7,376,669
Average length of lactation (days)	300	346.69	342.14	333.45	324.21
	350	370.61	359.31	343.01	329.57
	400	388.81	365.62	339.98	330.51
Average daily milk yield/cow (kg)	300	21.354	21.617	22.142	22.734
	350	20.042	20.627	21.555	22.387
	400	19.115	20.267	21.736	22.318

**Table 5:** Development of data on herds that have undergone a successful insemination experiment as a function of success rate (20–50%) and milk production decline (300 days–400 days).

	Day	Successful Insemination Experiment			
		20%	30%	40%	50%
Number of cows in lactation	300	768	888	949	979
	350	841	929	971	989
	400	866	960	987	995
Total amount of milk (kg)	300	5,694,504	6,568,803	7,007,842	7,216,165
	350	6,259,145	6,891,374	7,180,072	7,294,644
	400	6,443,755	7,113,913	7,291,722	7,337,053
Average length of lactation (days)	300	360.80	347.47	335.24	324.73
	350	383.93	363.82	344.29	329.91
	400	401.40	367.32	339.49	329.65
Average daily milk yield	300	20.550	21.296	22.026	22.698
	350	19.377	20.381	21.477	22.364
	400	18.536	20.173	21.7611	22.368
Average calving intervals (days)	300	426.24	412.84	400.55	389.90
	350	439.78	425.16	407.62	394.21
	400	452.10	429.07	404.07	395.03
Milk yield calculated for the days between two calving	300	17.395	17.924	18.435	18.904
	350	16.916	17.441	18.140	18.716
	400	16.458	17.270	18.283	18.666

## Data Availability

The data presented in this study are available on request from the corresponding author. The data are not publicly available due to the fact that it was obtained from dairy farmers and we do not have their consent for publishing.
